# Cell Mechanics in Cancer: Integrating Mechanotransduction Pathways Within the Tumor Microenvironment

**DOI:** 10.1002/jcp.70184

**Published:** 2026-05-11

**Authors:** Merve Sevgi, Yağmur Işık, Caner Karaca, Esmahan Çağlar, Hasan Berkay Abdioğlu, Ferican Zendel, Yasemin Başbınar, Hüseyin Üvet

**Affiliations:** ^1^ Department of Bioengineering Yıldız Technical University Istanbul Turkey; ^2^ Department of Mechatronics Engineering Yıldız Technical University Istanbul Turkey; ^3^ Oncology Institute Dokuz Eylül University Izmir Turkey; ^4^ Department of Biotechnology, Institute of Science Istanbul University Istanbul Turkey; ^5^ Artificial Intelligence Research and Application Center Istinye University Istanbul Turkey

## Abstract

Single‐cell mechanical properties such as stiffness, elasticity, and viscosity, are crucial in governing biological processes like migration, proliferation, and differentiation. In cancer, the mechanical properties of cells undergo significant alterations, which contribute to tumor growth, metastasis, and resistance to therapy. This review focuses on cancer cell stiffness and explores how its regulation is disrupted by the complex interplay among cytoskeletal remodeling, nuclear mechanics, and extracellular matrix (ECM) interactions. Cancer‐associated fibroblasts (CAFs) and ECM composition within the tumor microenvironment (TME) modulate cellular mechanics via mechanotransduction pathways involving Yes‐associated protein/transcriptional coactivator with PDZ‐binding motif (YAP/TAZ) and integrin‐focal adhesion kinase (FAK) signaling. Increasing evidence supports cell stiffness as a promising diagnostic and prognostic biomarker, as well as a predictor of treatment response. Therefore, advanced techniques for measuring cell stiffness such as atomic force microscopy (AFM), Brillouin microscopy, and acousto‐holography are evaluated with a focus on their potential clinical applicability. However, translation into routine oncology practice remains limited by technical variability, lack of standardized protocols, and the need for large‐scale clinical validation. This review highlights the potential of integrating biomechanical markers into clinical workflows as a means to advance cancer diagnostics and enable more personalized therapeutic strategies.

## Graphical abstract

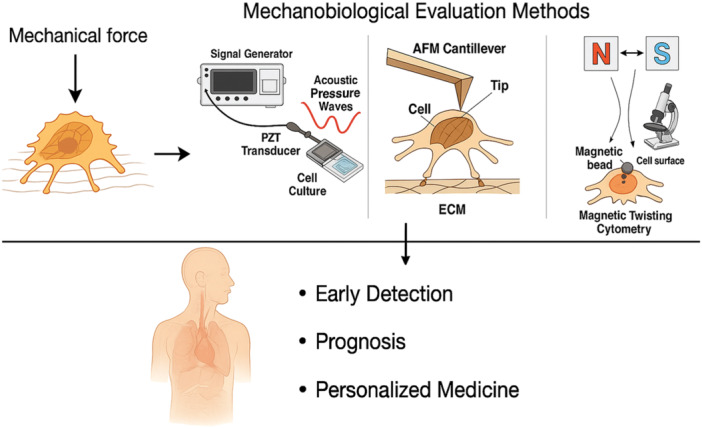

## 1 Introduction

The mechanical properties of single cells, such as stiffness, elasticity, and viscosity, are critical determinants of cellular behavior and function. They influence tissue development and regulate critical aspects of cancer pathology, including migration, differentiation, proliferation, and responses to external forces (Urbanska and Guck 2024; Rebelo et al. 2013; Quan and Kim 2016). Specifically, the viscoelastic nature of the microenvironment plays a decisive role in cell fate, a concept extensively explored by Chaudhuri et al. (2018) (Chaudhuri et al. 2018). Through continuous sensing and adaptation to mechanical stimuli, a process involving ancient complex mechanotransduction pathways that drive tumor progression (Nguyen and Farge 2024), cells interact dynamically with their microenvironment. These interactions are vital in both physiological and pathological contexts, underscoring the growing importance of cell mechanics in mechanobiology and cancer research (Yan 2024; Mierke 2024).

At the crossroads of physics and biology, mechanobiology unravels how mechanical signals are sensed and interpreted by biological entities like cells, tissues, and organs, and how these signals influence disease progression (Kim 2021). In this context, the seminal work of Weaver et al. (1997) demonstrated for the first time that the mechanical microenvironment can directly control the invasive phenotype of cancer cells. This study showed that by modifying extracellular matrix (ECM) stiffness and integrin signaling in three‐dimensional cell culture, the malignant phenotype of breast cancer cells could be reverted to a near‐normal state (Weaver et al. 1997). In cancer, both these mechanical signals and cellular structure change significantly, contributing to tumor progression, metastasis, and resistance to therapy. Therefore, changes in mechanical properties often serve as indicators of disease progression, particularly in aggressive conditions such as cancer (Liu et al. 2020; Alibert et al. 2017; Xu et al. 2012; Daniel et al. 2023; Han et al. 2020).

These alterations are strongly influenced by the tumor microenvironment (TME), a complex niche that modulates cytoskeletal organization through dynamic ECM composition and mechanical forces (Zhao et al. 2023; Espina et al. 2023).

Measuring cellular mechanics has long been challenging due to cells’ viscoelastic nature, which shows both solid‐like and fluid‐like behaviors depending on time scale and force type. However, recent technological advances have greatly improved the ability to characterize these properties (Papavassiliou et al. 2023). This review therefore synthesizes current insights into the role of cell stiffness in cancer, critically evaluates available methodologies, and discusses future directions for integrating biomechanical markers into oncology.

Despite the growing importance of mechanobiology in cancer research, a fundamental paradox remains unresolved. There is a notable discrepancy between findings suggesting that cancer cells become stiffer to gain metastatic potential and those proposing that they soften to adapt and survive during invasion and microenvironmental remodeling. The conflicting results may stem from differences in measurement techniques, cell cycle phases, or the contextual state of cellular components. Furthermore, the lack of standardized methods for assessing mechanical properties and translating these measurements into clinical practice remains a major limitation.

In this critical review, the existing literature will be briefly summarized, and this central contradiction in mechanobiology will be examined in depth. The review aims to identify the key signaling pathways that influence cell stiffness and mechanobiology, and to elucidate how the tumor microenvironment integratively regulates these pathways. In the following sections, the relationship between the cytoskeleton and cell stiffness will first be discussed, followed by an in‐depth analysis of the role of the tumor microenvironment in cellular mechanics. Finally, a comparison of stiffness measurement techniques will provide perspectives on current clinical challenges and future research directions.

## 2 Molecular Components of Cancer Cell Mechanics

Cancer is characterized by uncontrolled cell proliferation, leading to tumor formation, tissue invasion, and potential metastasis that disrupts organ function (Brown et al. 2023; Tafazzoli‐Shadpour et al. 2020). It exhibits hallmarks such as growth factor independence, immune evasion, metabolic reprogramming, and resistance to cell death. Additionally, cancer cells undergo biomechanical alterations involving cytoskeletal and ECM remodeling, which modify mechanical properties and promote migration and metastasis‐key drivers of cancer lethality.

The metastatic cascade begins with EMT, through which epithelial cells lose polarity and intercellular adhesion to acquire mesenchymal motility and invasiveness (Yilmaz and Christofori 2009). This phenotypic transformation enables cancer cells to degrade the ECM and penetrate the basement membrane by secreting matrix metalloproteinases (MMPs), thereby facilitating their entry into the surrounding connective tissue (Kim et al. 2015). Mesenchymal‐like cells then form focal adhesions via integrins and migrate through the interstitial matrix (Makale 2007). Within the tumor microenvironment, they disrupt nearby cells, evade immunity, and are guided by growth factors, cytokines, and chemokines toward blood vessels. There, they adhere to endothelial cells, secrete MMPs, increase endothelial permeability, and enter the bloodstream through gaps formed by endothelial contraction using amoeboid movement (Chiang et al. 2016).

Within the bloodstream, cancer cells soften to withstand hydrodynamic stress, evade immunity via platelet coating, resist apoptosis, and form microemboli before re‐adhering to endothelium through selectins and integrins (Sznurkowska and Aceto 2022; Liu and Cao 2016).

Extravasation requires mechanical flexibility for endothelial transit, followed by genetic and metabolic reprogramming, immune evasion, and niche construction at the secondary site to form distant metastases (Miles et al. 2008).

Another significant difference between cancer cells and healthy cells lies in the mechanical properties of the cell nucleus. The nuclei of cancer cells are typically larger, irregularly shaped, contain more DNA, are genetically unstable, have more permeable nuclear envelopes, and are more flexible and softer (Denais and Lammerding 2014). The presence of excess DNA makes cancer cells genetically unstable, which in turn increases their metastatic potential. These mechanical properties are critical in terms of cancer formation, progression, and metastasis.

### 2.1 Cytoskeleton

**Figure 1:**
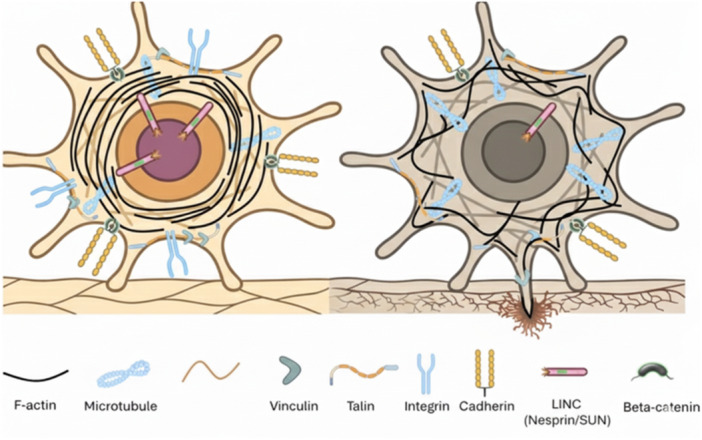
Comparative schematic representation of a non‐malignant (left) and a malignant cell (right) illustrating cytoskeletal organization and cell adhesion components. The cytoskeleton consists of Filamentous actin (F‐actin) filaments, microtubules, and intermediate filaments, each contributing to cell shape, polarity, and mechanical stability. Cell–cell and cell–matrix interactions are mediated by cadherin in adherens junctions and integrin in focal adhesions. Vinculin and talin serve as key adaptor proteins linking integrins to the actin cytoskeleton, facilitating mechanical force transmission and signaling. The malignant cell demonstrates significant remodeling, characterized by the downregulation of the Linker of Nucleoskeleton and Cytoskeleton (LINC) complex and focal adhesion proteins (vinculin/talin), alongside a loss of microtubule polarity. Furthermore, the disruption of adherens junctions triggers the release and nuclear translocation of β‐catenin, shifting its role from a structural stabilizer to a potent transcriptional co‐activator of epithelial‐mesenchymal transition (EMT)‐related genes. These structural changes, including the transition toward a more disorganized F‐actin network, facilitate increased nuclear deformability and the formation of invadopodia, ultimately driving the aggressive, metastatic phenotype associated with cancer progression (Di 2023).

The cytoskeleton is a fibrous structure composed of three main structural components: microfilaments, intermediate filaments, and microtubules (Bershadsky and Vasiliev 2012). It plays a crucial role in regulating intracellular organization, the cell's connection with the external environment, cell movement, and cell shape (Fletcher and Mullins 2010). In addition to these processes, the cytoskeleton is also involved in cell division, intracellular transport, and signal transduction (Bershadsky and Vasiliev 2012). As shown in Figure 1, the cytoskeleton in cancer cells undergoes structural alterations to enable processes such as uncontrolled division and shape changes, which distinguish them from normal cells. These modifications allow cancer cells to adapt to their invasive and metastatic behaviors, facilitating their ability to migrate, invade surrounding tissues, and survive in diverse microenvironments.

#### 2.1.1 Actin Cytoskeleton and Cellular Mechanical Sensitivity

Living cells sense mechanical cues from the extracellular milieu and integrate these into intracellular biochemical signaling cascades, a process termed mechanotransduction, with the actin cytoskeleton playing a central role (Jégou and Romet‐Lemonne 2021). Owing to its polarized and semi‑flexible filamentous architecture, actin generates the mechanical forces required for both cell motility and morphological remodeling (Hohmann and Dehghani 2019). Actin exists in two forms: Globular (monomeric) actin (G‑actin) and filamentous actin (F‑actin). In particular, F‐actin constitutes a key structural determinant of cellular stiffness. Recent studies have revealed a strong correlation between actin filament organization and cell stiffness. For instance, pathogenic protein aggregates such as Huntingtin protein (Q138 polyglutamine variant) (HTT Q138) and TAR DNA‐binding protein 43 (TDP‑43) disrupt F‑actin structure, resulting in increased cellular rigidity (Singh 2024). The Adenosine triphosphate (ATP)‑dependent polymerization of G‑actin into F‑actin fuels protrusive structures at the cell front‐lamellipodia, filopodia, and blebs (Parlani et al. 2023). These cortical actin‐based extensions, coupled with actomyosin interactions, generate cortical tension that governs cell shape, polarity, motility, and overall mechanical properties (Li and Wang 2020). Crucially, the density and organization of actin filaments, together with their interaction with myosin II, dictate cellular stiffness, thereby linking cytoskeletal architecture to cell mechanics.

Disruption of the cytoskeleton plays a central role in various pathologies, including cancer progression (Aseervatham 2020). Aberrant expression or activation of actin‐regulating proteins facilitates invasion and metastasis. In this context, the RhoA/ROCK/LIMK–cofilin pathway—comprising the small guanosine triphosphatases (GTPases) Ras homolog family member A (RhoA), Rho‐associated protein kinase (ROCK), and LIM domain kinase (LIMK)—is critical for regulating cortical tension. Activation of RhoA leads to ROCK‐mediated phosphorylation of LIMK, which inhibits cofilin. Consequently, actin filaments become stabilized and cortical tension increases. This mechanism is essential for dynamic cellular events related to cancer progression, including EMT and cell migration (Li and Wang 2020).

In addition to extracellular mechanical cues, intracellular regulatory pathways and actin‐binding proteins (ABPs) critically govern actin polymerization and cytoskeletal remodeling. Actin filament nucleation, stabilization, cross‐linking, and depolymerization are tightly orchestrated by ABPs. The small GTPases RhoA, Ras‐related C3 botulinum toxin substrate 1 (Rac1), and Cell division control protein 42 (Cdc42) are key regulators of cytoskeletal reorganization (Vakhrusheva et al. 2022). Their activities are modulated by kinases and phosphatases, thereby enabling rapid cytoskeletal remodeling in response to both biochemical and mechanical stimuli.

#### 2.1.2 Actin‑Based Adhesion Mechanisms and ECM Interaction

The actin network provides mechanical support by simultaneously binding to transmembrane adhesion proteins along the plasma membrane. These proteins enable cells to sense extracellular chemical and mechanical cues and initiate corresponding intracellular responses (Wen and Janmey 2011). Among transmembrane adhesion proteins, integrins and cadherins are of paramount importance (Xie 2023).

Cadherins, primarily mediating cell–cell adhesion, maintain tissue mechanical integrity (Maître and Heisenberg 2013). Classical cadherins such as Epithelial cadherin (E‑cadherin) and Neural cadherin (N‑cadherin) engage in sequential interactions‐first with β‐catenin, then with α‐catenin‐ via their cytoplasmic tails, thereby linking to the actin cytoskeleton. This connection modulates cellular responses to mechanical force (Loh et al. 2019). Tensile stress applied to cells induces conformational alterations in α‑catenin, unveiling binding sites for mechanosensory proteins such as vinculin. Vinculin's recruitment to adhesion complexes promotes their maturation and triggers reorganization of actin filaments, culminating in significant changes in cellular stiffness (Charras and Yap 2018).

The mechanical functionality of cadherins is inherently calcium‐dependent. Binding of three calcium ions to the extracellular domains confers rigidity and stabilizes homophilic cell–cell interactions. In particular, two of these ions support structural stiffness at the cadherin–cadherin interface, ensuring sustained adhesion. Calcium deficiency leads to structural destabilization, weakening both adhesive strength and cellular mechanical integrity (Barcelona‐Estaje et al. 2021).

Integrins mediate cell–ECM interactions and form focal adhesions (FAs), which connect the ECM with the actin cytoskeleton (Pang 2023). Talin binds to the integrin cytoplasmic tail and undergoes conformational activation, exposing vinculin‐binding domains that strengthen FAs (Chorev et al. 2018). This activation promotes cell adhesion and spreading on the ECM (Goult et al. 2021). Integrin signaling also recruits paxillin and focal adhesion kinase (FAK), activating Rho GTPases and ROCK, which regulate actomyosin tension and cell stiffness (Sun et al. 2016).

Moreover, a complex signaling network termed “adhesive cross‑talk” orchestrates coordination between cell–ECM and cell–cell adhesion systems. This mechanism requires the concerted activation of both integrins and cadherins. These receptors serve as dynamic mechanosensitive sensors on the cell surface, playing shared roles in signal transmission, cytoskeletal linkage, and regulation of cell behavior (Barcelona‐Estaje et al. 2021).

In cancer cells, alterations in adhesion structures are closely associated with metastatic behavior. Loss of E‐cadherin is a hallmark of epithelial transformation and facilitates dissemination, while EMT‐induced N‐cadherin expression exacerbates adhesion weakening (Loh et al. 2019). These reorganizations impact actin cortex structure, reducing stiffness and enhancing invasiveness.

#### 2.1.3 Intermediate Filaments and Mechanical Plasticity

Intermediate filaments (IFs) serve as key structural components that confer mechanical support and resilience to cells, particularly under bending and tensile stress (Köster et al. 2015; Cooper 2000). Vimentin plays a critical role in regulating cellular mechanical behavior through its multifaceted interactions with signaling pathways and cytoskeletal structures.

Notably, a bidirectional interaction exists between vimentin and Rac1. Vimentin modulates Rac1 activation, while Rac1, in turn, induces the phosphorylation of vimentin filaments. This reciprocal regulation facilitates the dynamic remodeling of the cytoskeleton, influencing cell shape and migratory capacity. In the absence of vimentin, both Rac1 and Cdc42 activity levels are diminished, leading to impaired cellular mechanical responses. Additionally, vimentin indirectly regulates FA organization by influencing integrin activation. Thick vimentin filaments are typically associated with stabilized FAs, whereas vimentin depletion correlates with mechanically unstable adhesions (Ostrowska‐Podhorodecka and McCulloch 2021). This multilayered interaction network underscores the pivotal role of vimentin not only in maintaining cellular architecture but also in modulating stiffness and mechanosensory responses to the extracellular environment.

Vimentin expression is markedly elevated in both primary and metastatic tumors, which correlates with poor prognosis. Beyond facilitating EMT, vimentin reorganizes the tumor microenvironment. With high tensile strength and resistance to mechanical stress, vimentin enables cells to breach the basement membrane (Ridge et al. 2022). Collectively, vimentin orchestrates cell mechanics and migratory dynamics, positioning it as a critical player in tumor progression and metastasis.

#### 2.1.4 Mechanical Integration and Stability Through Microtubules

Microtubules are the most rigid elements of the cytoskeleton and play essential roles not only in cell shape, polarity, and motility but also in maintaining and modulating cellular mechanics (Matis 2020). According to the tensegrity model, microtubules act as compressive elements that counterbalance tensile forces within the cell.

Structurally, microtubules are cylindrical polymers composed of 13 protofilaments made up of α‐ and β‐tubulin heterodimers (Amos and Hirose 2007). The helical arrangement of tubulin subunits supports the directed movement of motor proteins along the microtubule lattice, facilitating intracellular transport and mitosis. Due to dynamic instability, the plus ends (β‐tubulin‐exposed) of microtubules exhibit rapid polymerization and depolymerization, enabling them to function as flexible, highly responsive elements (Amos and Klug 1974).

Through interactions with with various microtubule‐associated proteins (MAPs), microtubules gain stability and promote bundled arrays (Hawkins et al. 2010). Connectivity with actomyosin enables resistance to tensile stress, thereby contributing to stiffness modulation under mechanical stress.

Microtubules also influence cancer cell invasiveness by coordinating with actin dynamics. ECM stiffness can induce post‐translational modifications of microtubules such as acetylation and glutamylation, increasing stability. Acetylated microtubules facilitate polarity and invasiveness, while destabilization activates the RhoA/ROCK axis, elevating cortical stiffness (Legátová et al. 2023). Thus, beyond their canonical roles in intracellular transport and mitosis, microtubules critically reprogram the mechanical state of cancer cells.

#### 2.1.5 The Central Role of Focal Adhesion Kinase (FAK) in Cellular Mechanics

FAK functions as a critical integrator of mechanical signals derived from interactions between the cell and the ECM (Cheng 2020). FAK plays a pivotal role in the assembly and disassembly of FAs, thereby orchestrating cell migration. Upon activation, FAK undergoes autophosphorylation at tyrosine 397 (FAK^Y397), creating a high‐affinity binding site for the Src homology 2 (SH2) domain of Src family kinases. Subsequent binding of Src leads to additional FAK phosphorylations, which trigger downstream activation of signaling proteins such as Grb2, p130Cas, and phosphoinositide 3‐kinase (PI3K), ultimately resulting in extracellular signal‐regulated kinase (ERK) activation (Xie 2023).

A mathematical model has demonstrated that clusters of integrins can convert ECM stiffness cues into FAK^Y397 phosphorylation events, effectively allowing FAK to function as a mechanical sensor that translates physical information into intracellular responses. FAK further transmits these mechanical cues to the actin cytoskeleton by activating Rho GTPases (RhoA, Rac1, and Cdc42) and downstream ROCK signaling pathways, which are central regulators of actomyosin tension (as detailed in Section 2.1.1) (Li and Wang 2020; Kalli et al. 2022). Notably, RhoA‐mediated activation of ROCK enhances actomyosin contractility, leading to the formation of stress fibers (Chauhan et al. 2011).

The upstream mechanical regulators of the Hippo signaling pathway‐including cytoskeletal tension, cell–cell and cell–matrix interactions, cellular morphology, and cell density‐critically influence the activity of the pathway (Mokhtari et al. 2023). The core effectors of Hippo signaling, Yes‐associated protein (YAP) and transcriptional coactivator with PDZ‐binding motif (TAZ), sense mechanical inputs from the extracellular matrix and cytoskeleton to modulate pathway activity (Seo and Kim 2018). FAK has been identified as a key modulator of YAP/TAZ nuclear translocation and transcriptional activity, thereby contributing to mechanotransduction through the Hippo axis (Holland et al. 2024; Lachowski et al. 2018).

Microtubules extending toward the cell periphery interact with focal adhesions through regulatory proteins, particularly via the KANK1–talin interaction. Microtubule targeting of adhesion sites locally inhibits GEF‐H1, thereby transiently suppressing RhoA activity. This leads to reduced actomyosin contractility and a decrease in traction forces, ultimately resulting in focal adhesion disassembly, accompanied by FAK dissociation from these structures (Aureille et al. 2024). However, the precise contribution of FAK to microtubule‐mediated adhesion turnover remains to be fully elucidated.

Additionally, while vimentin does not directly bind integrins, it contributes to integrin‐mediated mechanotransduction through its regulatory effects on FAK signaling, membrane tension, and RhoA activity (Jiu et al. 2017). These multifaceted interactions position FAK as a central integrator of mechanical signals within the cytoskeletal network, playing a fundamental role in determining cellular mechanical properties.

#### 2.1.6 The “Stiffer and Softer” Paradox in Cancer Mechanobiology

Detailed research in mechanobiology has provided compelling evidence that cellular stiffness often decreases during invasion and metastasis (Mierke 2025). However, literature also demonstrates that, depending on the biological context and cell state, increased myosin activation by stiffness can lead to increased cellular rigidity (Samuel et al. 2011). Furthermore, various 3D studies report that the tumor as a whole (bulk tissue) is significantly stiffer than normal tissue due to increased collagen cross‐linking and stromal cell activation (Sun 2021; Levental et al. 2009). This apparent contradiction raises critical questions regarding the reliability and applicability of mechanical biomarkers in clinical settings. To analyze the “stiffer and softer paradox” in cancer, it is essential to distinguish between the scale of measurement, the technical parameters of the methodology used, and the specific biological stage of the cell.

I. **Scale‐Dependent Divergence: Bulk Tissue Versus Single Cell**

A fundamental paradox in cancer mechanics stems from the divergence across biological scales.

- **Macroscopic Scale (Tissue Stiffness):** In healthy tissue, stromal cells synthesize ECM components and sense matrix stiffness to regulate remodeling, maintaining a state of homeostasis. In tumor tissue, dysregulated biological processes disrupt this ECM homeostasis (Sun 2021). Tumor tissue becomes stiffer than normal tissue due to increased collagen density and ECM cross‐linking. This desmoplastic stiffening results from the reciprocal interaction between cancer‐associated fibroblasts (CAFs) and the ECM. The stiffened matrix activates factors such as YAP and Twist1, creating a positive feedback loop that enhances CAF contractile capacity and matrix component synthesis. Enzymes of the lysyl oxidase (LOX) and LOX‐like (LOXL) families, triggered by transforming growth factor beta (TGF‐β) and hypoxia (hypoxia‐inducible factor 1‐alpha, HIF‐1α), govern collagen cross‐linking to reinforce this stiff mechanical barrier (Zhang and Zhang 2025).
- **Microscopic Scale (Cellular Softness):** The conventional “adaptive softening hypothesis” posits that cancer cells reduce their cytoskeletal stiffness to facilitate migration through the dense ECM and narrow tissue pores (Mierke 2025). As detailed in Section 2.1, vimentin accumulation and actomyosin network reorganization during the EMT reduce cortical tension to enable this transition. Literature consistently shows that many cancer cells are softer than their healthy counterparts (Massey 2024). Atomic force microscopy (AFM) and optical stretching measurements have demonstrated that bladder, breast, cervical, colorectal, and thyroid cancer cells exhibit significantly lower stiffness, which correlates with increased metastatic capacity at the population level (Mierke 2025). However, recent studies suggest that within a tumor population, a specific subpopulation of cells may undergo even further softening compared to their neighbors to enhance deformability. Conversely, this softening is not universal; leukemia, hepatocellular carcinoma, and chondrosarcoma cells have been found to be stiffer. In chondrosarcoma, increased stiffness is linked to high β‐tubulin expression, proving that the adaptive softening hypothesis is context‐dependent (Mierke 2025). Thus, while “cancer stiffens” describes the macroscopic tissue state, “cancer softens” typically refers to microscopic cellular adaptation, though notable exceptions exist even at the single‐cell level.

II. **Methodological Influence**

The apparent stiffness of a cancer cell is heavily influenced by the measurement modality. Comparative studies on identical cell lines have revealed that reported elastic moduli can vary by orders of magnitude depending on the technique employed (Wu et al. 2018). This discrepancy, which contributes significantly to the “stiffer versus softer” paradox in mechanobiology, primarily stems from three physical biases:

- Probing Depth and Scale: Surface‐based methods with sharp nanoscale probes typically measure the local tension of the actin‐rich cortex, often yielding stiffer readouts. In contrast, whole‐cell deformability assays average the composite response of the softer cytoplasm and the nucleus, frequently reporting a softer phenotype for the exact same cell state (Wu et al. 2018).
- Loading Rate (Viscoelasticity): Because cells are viscoelastic, the rate of applied force dictates the readout. Rapid force application (high frequency) captures a solid‐like, stiffer response, whereas slow deformation allows for viscous energy dissipation, yielding a softer profile (Hecht et al. 2015; de Sousa et al. 2020).

Substrate‐Induced Pre‐stress: Cells cultured as adherent monolayers on rigid 2D plastic develop artificial cytoskeletal tension (pre‐stress) via robust focal adhesions, appearing much stiffer. In contrast, cells evaluated in suspension or within compliant 3D matrices lack this artificially induced tension, revealing a softer baseline that more accurately mimics physiological migration (Discher et al. 2005; Solon et al. 2007; Pelham and Wang 1997).

III. **Stage‐Specific Adaptations**

Cellular mechanics exhibit distinct stiffness profiles across the metastatic cascade, driven by dynamic adaptations to changing microenvironmental conditions.

- **Early Invasion and Proliferation:** High compressive stress within the primary tumor creates a mechanical selection pressure. One hypothesis suggests that stiffer, less deformable cells may be preferentially ejected from the epithelium. In contrast, other models argue that cells at the invasive front soften via the EMT program or water pressure differentials to actively depart from the primary tumor (Gensbittel et al. 2021).
- **3D Migration and Nuclear Mechanics:** During interstitial migration, adaptability is more critical than absolute softness. A soft nucleus provides a passive advantage when passing through restrictive pores, while the process of amoeboid migration requires the cell to actively and continuously adapt its shape to the immediate environment (Gensbittel et al. 2021).
- **Intravasation and Extravasation:** These stages involve severe mechanical challenges requiring significant deformation to pass through endothelial gaps. However, this is not always a purely active cellular deformation; tumor cells can disrupt endothelial wall integrity or induce endothelial remodeling, allowing them to transit with minimal shape change (Gensbittel et al. 2021). Circulating Tumor Cells (CTCs) and Fluid Shear Stress (FSS): CTCs encounter fluid shear stress in the bloodstream. While some studies suggest that softness is required to survive FSS, others demonstrate that RhoA and Myosin II‐mediated stiffening enhances CTC survival rates by providing mechanical resilience (Gensbittel et al. 2021).
- **Dormancy and Resistance:** In the dormant state, cells may exhibit a stiffer profile to remain resilient against physical and chemical stressors. For instance, Polyploid Giant Cancer Cells (PGCCs), which are key representatives of dormant populations, display high cytoplasmic and nuclear stiffness to resist chemical stress and chemotherapy (Xuan et al. 2018) Conversely, if the primary goal is to evade immune cells such as cytotoxic T lymphocytes (CTLs) and natural killer (NK) cells, dormant cells may adopt a softer profile (Gajda et al. 2025).
- **Mechanical Consequences of EMT, Immune Evasion, and Checkpoints:** Beyond metastatic progression, mechanical properties offer an immunological advantage. Stiffer tumors may exhibit lower immune cell infiltration. Furthermore, immune cell activity is modulated by target cell mechanics; softer cancer cells can weaken the cytotoxic attacks of lymphocytes, acting as a mechanical immune checkpoint (Mierke 2025).

## 3 Mechanical Changes in the Cancer Microenvironment

The TME is a complex structure composed of tumor cells, stromal cells, tumor microvessels, various immune cells, CAFs, and the non‐cellular components within the ECM (Spill et al. 2016). During tumor progression, various physicochemical properties of the TME change (Shah et al. 2022). These changes can have a direct effect on the stiffness of cancer cells.

As a result of the rapid proliferation of cancer cells, solid stress accumulates during tumor progression. The solid stress accumulated during tumor progression is divided into two main types (Jain et al. 2014; Hagendoorn et al. 2006; Stylianopoulos et al. 2012). The first is stress due to cell proliferation, which arises from microscopic interactions between cells in the TME and stromal components and remains in the tumor even if external loads are removed (Stylianopoulos et al. 2012). The second is externally applied stress, which is generated by the host tissue to inhibit tumor expansion and decreases when the tumor is excised (Cheng et al. 2009; Demou 2010). During tumor formation, solid stress and extracellular matrix stress interact as the external and internal forces of the tumor, respectively. Solid stress refers to the tension accumulated in the solid phase of the tumor, and as the tumor grows, it is the mechanical pressure exerted by the surrounding normal tissue to limit tumor expansion (Chen et al. 2019; Stylianopoulos et al. 2013). The proliferation of tumor cells and the increase in tumor volume enhance solid stress exerted by surrounding tissues. This condition leads to the compression of tumor vasculature, tissue hypoxia, stimulation of local inflammatory responses, and activation of CAFs (Melica et al. 2019; Wells 2008). Furthermore, tumor growth pressure exerts profound tumorigenic effects on the surrounding epithelial environment. Mechanical strain has been shown to activate oncogenic signaling pathways in neighboring healthy cells, effectively transforming the local stroma into a supportive niche (Benham‐Pyle et al. 2015; Fernández‐Sánchez et al. 2015) (demonstrated in cell culture (Benham‐Pyle et al. 2015) and in vivo (Fernández‐Sánchez et al. 2015)). Crucially, this mechanical induction modifies the microenvironment to promote and maintain cancer stem cell (CSC) production, as physical pressure serves as a critical driver of cellular stemness in vivo (Nguyen Ho‐Bouldoires et al. 2022). Cancer‐associated fibroblasts play a role in the synthesis of ECM components. Activated CAFs synthesize ECM, produce cytokines and chemokines, and exert physical forces, leading to local fibrosis, increased capillary pressure, local ischemia, hypoxia, and increased ECM stiffness (Biffi and Tuveson 2021; Parsonage et al. 2005; Kalluri 2016). Matrix metalloproteinases secreted by cancer‐associated fibroblasts degrade various protein components in the ECM, removing the histological barrier to tumor cell invasion and promoting tumor invasion and metastasis (Bonnans et al. 2014). The stiff tumor microenvironment resulting from ECM remodeling associated with the tumor is characterized by increased ECM accumulation, fiber alignment, and cross‐linking. These changes have been shown to actively promote tumor progression and malignancy through enhanced integrin signaling (Paszek et al. 2005). Additionally, plasmin, serine protease elastase, and cathepsins soften the ECM and promote the degradation of fibronectin and elastin (Smith and Marshall 2010; Bonnefoy and Legrand 2000).

The heterogeneity of cells forming the tumor is also among the factors affecting the microenvironment. To support their invasive growth, cancer cells attract various host cells within the TME. These cells include fibroblasts, angiogenic endothelial cells, and immune cells (Egeblad et al. 2010). The extracellular matrix is jointly produced and secreted into the extracellular space by tumor cells and host cells. During tumor progression, the composition, structure, and mechanical properties of the ECM undergo significant changes (Lu et al. 2012). Particularly in breast cancers, Type I collagen becomes highly linearized as if under tension and aligns either tangentially or perpendicularly to the tumor‐stroma interface (Provenzano et al. 2008).

The oxygen metabolism of cells and tumors leads to rearrangements by affecting signaling pathways. Hypoxia has been shown to activate the Notch signaling pathway, increasing the expression of Snail‐1 and LOX. This mechanism leads to rearrangements in the cytoskeleton, reducing cell stiffness while increasing matrix stiffness via LOX in the tumor microenvironment, thereby enhancing the invasion and metastasis potential of cancer cells (Sahlgren et al. 2008). Additionally, LOX strengthens integrin signaling and proliferation in the tumor matrix by cross‐linking Type I collagen and other fibrillar collagens (Barker et al. 2012). Mechanically, the secreted LOX is responsible for the invasive properties of hypoxic human cancer cells via FAK activity and cell‐ECM adhesion. A study showed that LOX inhibition delayed tumor formation and reduced incidence in ErbB2‐associated breast cancer models. Tumors became smaller, less proliferative, the myoepithelium was preserved, and the rate of premalignant lesions increased. These findings indicate that LOX inhibition reduces fibrosis by decreasing collagen cross‐linking, lowers focal adhesions, and prevents tumor progression (Levental et al. 2009).

ECM stiffening reshapes cellular mechanics through mechanotransduction pathways, increasing the invasion capacity of cancer cells. This process may lead tumor cells to acquire a metastatic phenotype by activating mechanosensitive effectors such as YAP/TAZ, which respond to extracellular stiffness through the actin cytoskeleton and focal adhesion points. Therefore, mechanical changes in the tumor microenvironment are critical not only in determining cell stiffness but also in regulating cell motility and plasticity (Gkretsi and Stylianopoulos 2018). A study has shown that YAP and TAZ are critical transcriptional regulators in sensing the physical microenvironment of cells. This mechanism operates parallel to the NF2/Hippo/LATS pathway and is regulated in a Rho‐dependent manner via the actomyosin cytoskeleton. YAP/TAZ activity is controlled by cytoskeletal tension depending on ECM stiffness and cell spreading, and this mechanism has been emphasized as playing a crucial role in cancer progression and the formation of metastatic niches (Dupont et al. 2011; Miralles et al. 2003; Jaalouk and Lammerding 2009). Furthermore, YAP activity plays a critical role in ECM stiffening and increased cancer invasion, which are supported by CAFs (Calvo et al. 2013). Since increased ECM stiffness is associated with increased YAP activity (Gkretsi and Stylianopoulos 2018), CAFs establish a positive feedback loop between cell stiffness and YAP activity (Calvo et al. 2013).

Integrins are well‐defined mechanoreceptors that physically link the actomyosin cytoskeleton to the surrounding matrix and transmit signals. The α and β subunits can form various heterodimers associated with cancer progression in different cancer types. These heterodimers serve as the central communication hub between cells and the tumor microenvironment (Jang and Beningo 2019). The integrin αvβ3 has been found to sense the stiffness of fibronectin‐coated surfaces. When fibroblasts were treated with an anti‐αvβ3 monoclonal antibody that blocked the interaction between integrin αvβ3 and fibronectin, they failed to sense the stiffness of the fibronectin matrix (Jiang et al. 2006). Similarly, cells deficient in receptor‐like protein tyrosine phosphatase (RPTPα) lost their ability to sense matrix stiffness. This finding suggests that integrin αvβ3, in conjunction with RPTPα, plays a role in sensing the stiffness of the fibronectin matrix at the leading edge during early cell spreading (Jang and Beningo 2019).

Genes encoding matrix proteins and remodeling enzymes are generally not deleted or amplified in primary cancers. Therefore, oncogene‐driven transcriptional changes, epigenetic reprogramming toward a less differentiated state, and EMT processes likely contribute together to the altered composition of the tumor matrix (Cooper and Giancotti 2019).

Another study showed that cancer cells have lower stiffness compared to normal cells and are insensitive to changes in matrix stiffness. This mechanical adaptation has been associated with the suppression of Caveolin‐1 protein, and restoring Caveolin‐1 expression has led to the normalization of mechanical properties in transformed cells. These findings suggest that Caveolin‐1 may influence invasion and progression by regulating cellular mechanical properties in the tumor microenvironment (Lin et al. 2015).

The tumor microenvironment plays a crucial role in cancer progression. Taken together, although tumors are generally stiffer than healthy tissues (Paszek et al. 2005), cancer cells themselves are usually softer than their normal counterparts (Cross et al. 2007)—a scale‐dependent divergence further examined in Section 2.1‐6.

### 3.1 Effects of Tumor‐Associated Fibroblasts on Cell Stiffness

**Figure 2:**
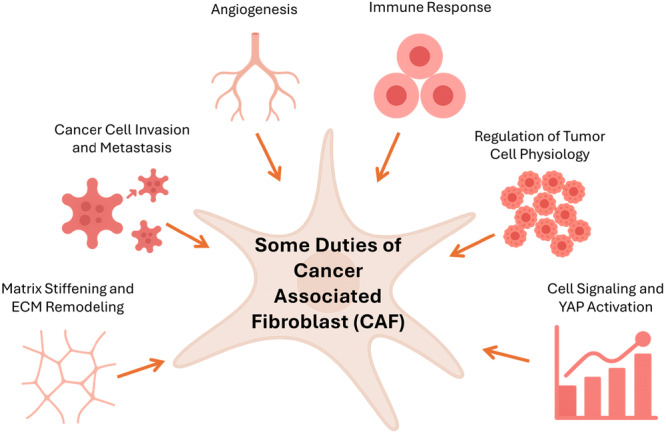
Functional roles of cancer‐associated fibroblasts (CAFs). This figure summarizes key functions of CAFs in the tumor microenvironment, including ECM remodeling, promotion of tumor cell invasion, secretion of growth factors and cytokines, immune modulation, and stimulation of angiogenesis.

Fibroblasts—both within and around the tumor—are key stromal cells that contribute to ECM production and tissue structure (Zhao et al. 2023; Zhou et al. 2022; Shiga et al. 2015). In the tumor context, they are referred to as cancer‐associated fibroblasts (CAFs). As shown in Figure 2, CAFs serve multiple significant functions (Sahai et al. 2020). CAFs are involved in the synthesis and accumulation of ECM components. Additionally, they reshape the ECM, impacting cancer cell invasion, metastasis, and angiogenesis. By releasing various signaling molecules, such as growth factors, cytokines, and chemokines, CAFs influence cancer cell proliferation. They can also interact with immune cells in the TME, modulating leukocyte functions and suppressing immune responses, thereby contributing to tumor immunology (Sahai et al. 2020). Furthermore, CAFs promote matrix stiffening and angiogenesis through YAP signaling (Calvo et al. 2013). Given their abundance in the TME and their critical role in metastasis, CAFs have been extensively studied. Therapeutic strategies focusing on the mechanical properties of CAFs could be developed to target the stiffness changes within the cancer microenvironment.

Cells within the TME are exposed to both internal and external forces (Zhou et al. 2022). In response to these mechanical forces, cells detect them through mechanosensing processes and convert these signals into biochemical ones via mechanotransduction pathways (Jaalouk and Lammerding 2009). This response to mechanical forces results in changes in cell adhesion (Yeung et al. 2005), morphology (McKenzie et al. 2018), motility (Mierke 2020), and migration (Espina et al. 2022; Mierke 2019). CAFs exert mechanical stress and deformation on cancer cells by compressing them (Calvo et al. 2013). As a result of this compression, changes occur in the stiffness of cancer cells. This is primarily due to CAFs’ compression forces being dependent on actomyosin contraction, with myosin IIA knockout CAFs applying less traction compared to control CAFs (Barbazan et al. 2023). Furthermore, inhibiting myosin IIA contraction using the blebbistatin inhibitor significantly reduces the compressive capacity of CAFs, demonstrating the critical influence of myosin II on intracellular tension and, consequently, cell stiffness (Barbazan et al. 2023; Nguyen et al. 2022a).

It has also been observed that the traction forces exerted by CAFs and intestinal fibroblasts are higher than those of murine embryonic fibroblasts. In particular, the high traction exerted by CAFs may increase cancer cell stiffness by compressing them. Additionally, CAF‐induced compression leads to a reduction in nuclear area and nuclear deformation in cancer cells. Consequently, the YAP exits the nucleus (Barbazan et al. 2023). Nuclear deformation is an essential parameter affecting cell stiffness, and this process induces significant changes in cellular mechanics (Rao et al. 2021).

Research indicates that Syndecan‐4, expressed by CAFs, collaborates with integrins in mechanotransduction and mechanosignaling (Zeltz et al. 2022). Another study demonstrated a force‐dependent syndecan‐integrin crosstalk, where tension on Syndecan‐4 synergistically activated β1 integrins, initiating a mechanosignaling cascade and adaptive stiffening responses. Increased RhoA activity then led to cytoskeletal rearrangement, resulting in an increase in cell stiffness (Chronopoulos et al. 2020).

Further research shows that YAP is essential for CAF establishment and maintenance. CAFs utilize YAP to promote matrix stiffening, cancer cell invasion, and angiogenesis. YAP is activated due to ECM remodeling and changes in the actin cytoskeleton, typically in response to mechanical stress. Actomyosin function creates isometric tension within cells, leading to the formation of stiffer matrices. This process activates Src family kinases in focal adhesions, which are necessary for YAP activation. The matrix stiffness resulting from YAP activation reinforces the YAP pathway seen in CAFs (Calvo et al. 2013). More studies are needed to better understand the role of YAP in the tumor microenvironment and to develop therapeutic strategies accordingly.

### 3.2 The Effect of ECM Composition on Cancer Progression

ECM composition and organization—primarily collagen, elastin, fibronectin, and proteoglycans—determine tissue mechanical properties and cell behavior; stiffer ECM elicits higher traction forces and larger focal adhesions (Levi et al. 2020; Fu et al. 2018; Guimarães et al. 2020; Alcaraz et al. 2008; Baker et al. 2010).

Changes in the amount of ECM components affect tissue density and stiffness. Mammographic density has been reported to be positively correlated with breast cancer risk (Nazari and Mukherjee 2018). Matrix stiffening is also associated with an increased risk of cancer in fibrotic organs (Samarelli et al. 2021). While ECM stiffness in the brain, lungs, breast, or pancreas is typically below 1000 Pa, it can reach 4–10 kPa in tumors within these regions (Piersma et al. 2020). Matrix stiffening generates mechanical signals on stromal cells, parenchymal cells, premalignant cells, and cancer cells, triggering processes such as cell transdifferentiation, autophagy, EMT, cell migration, invasion, and metabolic reprogramming (Dong et al. 2019; Pankova et al. 2016).

Collagen is the most abundant component of the ECM structure and contributes the most to its mechanical properties (Wenger et al. 2007; Shoulders and Raines 2009). Elastin, along with collagen, is one of the most prevalent components of the ECM and is commonly found in organ structures. Although studies on other matrix structures are limited, collagen and elastin have been the primary focus in matrix stiffness research (Wagenseil and Mecham 2012). Cross‐links in collagen have been recognized as significant regulators of desmoplasia, and it has been suggested that the level and quality of ECM cross‐linking in a tissue may influence cancer risk (Levental et al. 2009). In desmoplasia or dense ECM accumulation, Type I collagen and ECM regulators accumulate in the tumor. This condition can cause cancerous tissues to become ten times stiffer and denser than healthy tissues (Kai et al. 2019). The presence of thick and aligned collagen fibers at the invasive front of the primary tumor has been associated with poorer patient prognosis in breast cancer and other cancers (Conklin et al. 2011; Drifka 2014).

A study investigating the relationship between ECM proteins and stromal stiffness found that collagen fibers and elastin fibers are associated with the stiffness of breast lesions (Xiao et al. 2016). Large aggregates of elastin fibers, a condition known as elastosis, have been frequently observed in the stroma of breast cancer (Kristensen and Karsdal 2016; Kadar et al. 2002; Martinez‐Hernandez et al. 1977). Another study evaluated the elastin fiber content of malignant breast lesions and fibroadenomas, examining whether a correlation existed between the elastin fiber content of lesions and the shear wave velocity (SWV) values measured via ultrasonography. The results indicated that a high elastin fiber score was significantly more common in malignant lesions (*n* = 61; 81.3%) (*p* = 0.001). Additionally, a positive correlation was found between SWV values and tissue elastin fiber scores (Toprak I. Aras et al. 2022). These findings highlight the crucial role of ECM composition and stiffness in cancer progression. Specifically, collagen and elastin fibers can alter the mechanical properties of the tumor microenvironment, influencing cell behavior, invasion capacity, and tumor aggressiveness. Increased ECM stiffness leads to the formation of mechanical signals that enhance cancer cell adaptation and metastatic potential. Therefore, the dynamic changes in ECM components and their mechanical properties should be considered critical targets both for understanding cancer biology and for developing therapeutic approaches.

### 3.3 Influence of Biophysical Cues From the Microenvironment on Cellular Behavior

Cells respond to mechanical cues such as forces, ECM geometry, and stiffness (Mitrossilis et al. 2009). These cues trigger a mechanical response resulting from the deformation of load‐bearing cellular structures, as well as biochemical signaling that drives phenotypic changes (Janmey and McCulloch 2007; Hoffman and Crocker 2009). The process by which cells sense mechanical forces and convert them into biochemical signals is known as mechanotransduction (Xin et al. 2023). Key components of mechanotransduction include membrane receptor activation, nuclear reorganization, and mechanosensitive transcription factors (Guo et al. 2024). This process typically occurs between the ECM and neighboring cells or in the cell's immediate surroundings. As detailed in Section 2.1‐2, integrins transmit ECM‐derived forces to the actin cytoskeleton through the talin–vinculin axis (Guo et al. 2024; Kang et al. 2024; Mykuliak et al. 2024). In addition to the integrin‐talin axis at cell‐ECM adhesions, β‐catenin serves as a critical mechanosensor, particularly at cell‐cell junctions. As a well‐established and long‐standing marker of tumorigenesis, β‐catenin responds to mechanical strain and tissue pressure, which can trigger its release from the cell membrane and subsequent nuclear translocation. This process activates downstream oncogenic signaling pathways, directly illustrating how mechanical cues drive malignant transformation (Fernández‐Sánchez et al. 2015). These proteins exhibit mechanosensitive properties (Röper et al. 2018).

Fluid mechanics is another significant factor affecting cell stiffness. Blood pressure, blood viscosity, and shear stress are examples of fluid mechanical forces (Wei and Li 2023). During cardiovascular development, shear stress is high; however, the loss of shear stress in capillaries can lead to cell apoptosis (Dahl et al. 2010). Cells experiencing shear stress show altered gene expression (Brooks et al. 2004). The glycocalyx, a structure rich in carbohydrates, covers vascular endothelial cells and influences capillary red blood cell filling and various vascular (dys)functions (Berg et al. 2006). It connects with the cytoskeleton, aiding in the transmission of mechanical information to the cell. In narrow capillaries, the glycocalyx plays a vital role as cells respond to shear stress (Dahl et al. 2010). These factors collectively influence the behavior of cells within the blood and the inner walls of blood vessels (Wei and Li 2023).

When cells are exposed to shear stress, the cytoskeleton undergoes reorganization (McCue 2004). Cells elongate and flatten in the direction of flow, with actin filaments aligning parallel to the flow direction (Dahl et al. 2010). Flow‐induced tension also results in reorganization of intermediate filaments (Davies et al. 2003). These cytoskeletal rearrangements lead to changes in cell stiffness (Dahl et al. 2010). Considering these factors, cell stiffness holds potential as a diagnostic marker for diseases like cancer. Future research into measuring cell stiffness in biopsies could provide insights into tumor stage and aggressiveness. The mechanical properties of cells have promising potential for integration into clinical diagnostic protocols.

### 3.4 The Mechanotransduction Cascade: From Plasma Membrane to Nuclear Transcriptional Reprogramming

**Figure 3:**
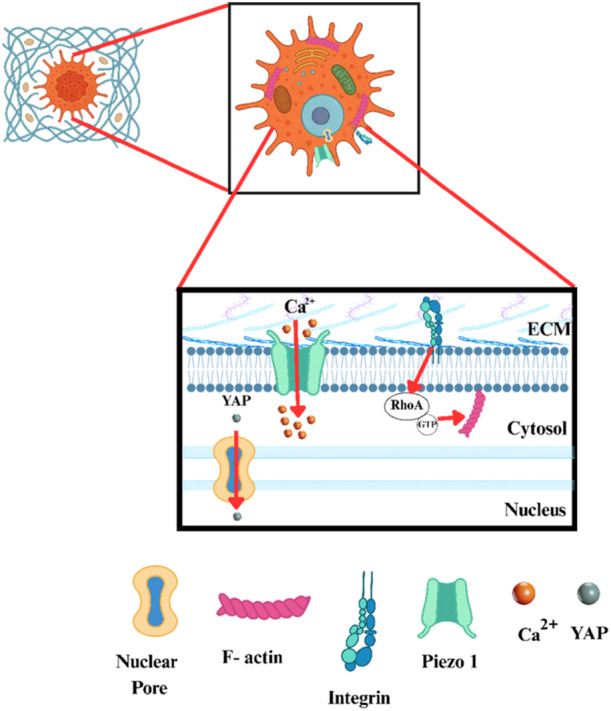
Mechanotransduction pathways regulated by the tumor microenvironment (TME). Schematic representation of key mechanotransduction pathways activated by the tumor microenvironment. Increased ECM stiffness enhances integrin clustering and FAK activation, which stimulates the RhoA/ROCK pathway to promote F‐actin stress fiber formation and cytoskeletal tension. These forces are transmitted to the nucleus via the LINC complex, facilitating nuclear translocation of YAP/TAZ transcriptional co‐activators that drive oncogenic gene expression and cellular reprogramming. In parallel, mechanosensitive ion channels such as Piezo1 rapidly respond to acute mechanical stress, inducing transient Ca²⁺ influx and actin remodeling that modulate short‐term cell stiffness. Together, these FAK/YAP/TAZ and Piezo1 pathways coordinate the mechanical adaptation of cancer cells to the TME, linking physical cues to biochemical signaling and transcriptional regulation.

The regulation of cancer cell mechanics is a tightly coordinated cascade that converts both mechanical and biochemical cues derived from the TME into functional cellular outputs (Shu et al. 2024). Cellular stiffness is governed by a complex feedback loop in which upstream mechanosensors (e.g., Piezo1, Integrins) and downstream effectors ‐such as FAK and YAP/TAZ‐ mutually reinforce one another to maintain mechanical homeostasis (Figure 3).

Mechanical signals are initially sensed at the cell surface by mechanosensitive ion channels such as Piezo1 and transient receptor potential vanilloid 4 (TRPV4), which rapidly trigger Ca²⁺ influx. Thus, the mechanotransduction pathway forms a continuous hierarchical axis from membrane sensors to nuclear effectors, directly linking physical cues in the tumor microenvironment to oncogenic transcription (Di 2023; Pang 2023; Liu 2022; Ueda 2022; Driscoll et al. 2015).

I. **Upstream Sensing via Mechanosensitive Ion Channels**
  Upstream mechanosensors, such as Piezo1 and other stretch‐activated ion channels, initiate the mechanotransduction cascade. Upon sensing mechanical stress and ECM stiffness, Piezo1 facilitates the influx of Ca²⁺. This calcium entry serves as a critical secondary messenger, priming the cellular machinery for subsequent mechanical adaptations (Liu 2022). In contrast to the sustained FAK/YAP/TAZ response, Piezo1 also facilitates rapid adaptation to acute mechanical changes within the TME; upon activation, the transient Ca²⁺ influx briefly modulates the actin cytoskeleton, resulting in a short‐term decrease in cellular stiffness and allowing the cell to respond instantaneously to fluctuating mechanical stimuli before the long‐term transcriptional adaptations take hold (Liu 2022).
II. **The Integrin‐Src‐FAK Complex and Adhesion‐Mediated Sensing**
  As TME stiffness increases, the elevated intracellular calcium levels promote integrin clustering. Consequently, cancer cells establish more robust adhesions to the ECM through these integrins. This engagement activates FAK in conjunction with Src family kinases. The synergistic FAK‐Src complex functions as a primary signaling hub, phosphorylating downstream targets that bridge the interaction between membrane‐bound receptors and the internal cytoskeleton (Pang 2023).
III. **The RhoA/ROCK Pathway and Actomyosin Tension**
  Following the activation of the Integrin‐Src‐FAK complex, signal amplification occurs through the RhoA/ROCK signaling axis. This pathway stimulates the assembly of F‐actin stress fibers and enhances actomyosin contractility. At this stage, the generated intracellular tension serves as a physical ‘transmission’ mechanism, ensuring the propagation of mechanical signals throughout the cell body (Di 2023).
IV. **Nucleo‐Cytoskeletal Coupling and YAP/TAZ Regulation**

A hallmark of profound mechanotransduction is the transmission of actomyosin‐generated tension to the nucleus via the LINC complex. Composed of Nesprin proteins in the outer nuclear membrane and SUN proteins in the inner nuclear membrane, the LINC complex directly transfers mechanical loads from the cytoskeleton to the nuclear lamina (Ueda 2022). This transmission modulates the organization of Lamin A/C, which determines the mechanical rigidity of the nuclear envelope. Furthermore, Lamin A/C senses mechanical stress that leads to the physical stretching and dilation of Nuclear Pore Complexes. This structural opening facilitates the nuclear translocation of YAP and TAZ transcriptional co‐activators from the cytoplasm (Driscoll et al. 2015). Once inside the nucleus, YAP/TAZ associate with TEA domain (TEAD) transcription factors to regulate the expression of genes involved in cancer cell growth, survival, and stemness. The FAK‐induced increase in stiffness further amplifies YAP/TAZ activity, leading to comprehensive phenotypic and transcriptional reprogramming. This transition represents a critical juncture where mechanical cues are converted into genetic outputs, ultimately consolidating the aggressive phenotype of the cancer cell (Dupont et al. 2011).

## 4 Cellular Stiffness as a Diagnostic and Prognostic Biomarker

Cancer cells dynamically remodel their mechanical properties during carcinogenesis including their cytoskeleton, nucleus, and plasma membrane, to secure a growth and survival advantage. As tumors become increasingly invasive, cells generally adopt a softer, more deformable phenotype that facilitates shape changes and enables them to traverse the rigid extracellular matrix as a critical adaptation for metastasis. In contrast, certain drug‐resistant cancer cell subpopulations have been found to exhibit increased stiffness, suggesting that the mechanical phenotype is heterogeneous and may reflect different adaptive strategies. These emerging insights into cellular stiffness not only enhance our understanding of cancer progression but also highlight the potential of biomechanical measurements as diagnostic and prognostic biomarkers.

Malignant cells tend to be softer than their normal counterparts, particularly in cancers of the breast, bladder, cervix, pancreas, and colon (Xu et al. 2012; Mykuliak et al. 2024; Tang et al. 2021a). During the multi‐step progression from normal to cancerous cells, mechanical properties are also governed by malignities in addition to genetic and biochemical processes. For example, studies on esophageal cell lines have demonstrated a decrease in Young's modulus from 4.7 kPa in normal EPC2 cells, to 3.1 kPa in metaplastic CP‐A cells, and further to 2.6 kPa in dysplastic CP‐D cells (Fuhrmann et al. 2011). Notably, the enhanced tumorigenicity of softer cells is also associated with an increased expression of stemness‐related genes such as *ABL1, COL1A1, JAK3*, and *BCL9L*, suggesting that intrinsic softness may be a unique marker for CSCs. In vivo experiments have further revealed that isolated soft tumor cells (< 400 Pa) possess significantly greater tumorigenic potential compared with their stiffer counterparts (> 700 Pa), as evidenced by their capacity to form tumors in the immunocompetent mice model. Therefore, even when cells exhibit stemness, mechanical properties were reported as selective biomarkers in tumor formation, suggesting soft subsets have more tumorigenic capacity than rigid counterparts (Lv 2021). Moreover, several oncogenes including *RAS, MEK, WNT*, and *BCL9* are reported correlated to cell softening (Tejeda‐Muñoz and Mei 2024). Considering these advancements, techniques that measure cell stiffness hold promise as diagnostic tools for detecting malignant transformation during carcinogenesis.

Metastatic cancer cells must deform and pass through the endothelium while resisting physical stress. To facilitate extravasation, this process is driven by actin cytoskeleton remodeling, bleb formation, and lamellipodia extension (Shi 2024). Soft tumor cells are often better adapted for amoeboid movement, a mode of migration that relies on low cellular stiffness. Some infamous oncogenes and ion channels also drive this adaptation (Lin et al. 2015; Lv 2021). Overexpression of mechanosensitive ion channel TRPV4 reduces the shear modulus by more than twofold, thereby lowering the force required for cell deformation and facilitating bleb formation. Likewise, the knockdown of TRPV4 results in fewer and less pronounced blebs (Lee et al. 2016). Furthermore, variations in cellular stiffness have been linked to organotropism; for instance, breast cancer cells with bone tropism exhibit increased stiffness and enhanced F‐actin organization, whereas those with brain tropism are generally softer (Tang et al. 2021a). Nevertheless, prostate cancer cells with aggressive metastatic phenotype are reported stiffer than their normal counterparts. This indicates that alterations in cell stiffness are an important step to metastasis but either softness or stiffness could serve different modes of metastasis (Molter et al. 2022). Therefore, the measurement of Young's modulus remains a promising parameter for cancer‐specific characterization of metastatic behavior and organotropism.

The ability of cells to detect and respond to mechanical cues is a fundamental process from the earliest stages of embryogenesis to the construction and differentiation of complex organs (Goodwin and Nelson 2021; Shroff et al. 2024). Considering cancer as a developmental anomaly, mechanosensing emerges as one of the most essential tumorigenic regulators (Papavassiliou et al. 2023; Cambria et al. 2024). During carcinogenesis, cancer cells lose the ability to sense matrix stiffness, which results in unhindered proliferation while reduced cellular stiffness supports progressive phenotype. Mechanosensing of the ECM stiffness depends on cytoskeletal and membrane adhesion to the matrix. Disruption of caveolin‐1, a co‐player to the mechanoreceptor integrin, causes cells to lose their ability to interact with the matrix while softening cytoskeletal stiffness, resulting in resistance to anoikis and anchorage‐independent growth (Lin et al. 2015). Calcium regulators and ion channels including stromal interaction molecule (STIM) proteins, transient receptor potential melastatin 7 (TRPM7), and TRPV4, also play an important role in orchestrating cytoskeletal reorganization, which leads to reduced cellular stiffness and facilitates metastatic processes such as amoeboid movement, enhanced invasiveness, and efficient extravasation (Lee et al. 2016; Chen et al. 2013). Understanding these mechanisms of the cellular transformation leads to potential therapeutic options to overcome physical responsiveness and the ability to progress.

Cell mechanics also influence therapeutic outcomes. Several studies indicate that treatment modalities, including chemotherapeutic agents, small molecule inhibitors, and radiotherapy may lead to an increase in cellular stiffness. For example, treatment with cetuximab has been shown to enhance the cytoskeletal intensity and stiffness of breast cancer cell lines such as MDA‐MB‐231 and MCF‐7 (Azadi et al. 2019). Furthermore, inhibition of epidermal growth factor receptor (EGFR) downstream targets, Ras and MEK1/2, recovers cellular probing to ECM and blocks malignant progression (Lin et al. 2015). Similarly, administration of 50 μM epigallocatechin gallate increased Young's modulus of metastatic lung cancer cell lines H1299 and Lu99 nearly twofold and reduced EMT (Takahashi et al. 2014). In prostate cancer, cells surviving docetaxel and cisplatin treatments exhibited higher stiffness, correlating with reduced motility and invasiveness (Raudenska et al. 2019). On the other hand, PGCCs, often associated with dormancy and relapse, also display higher cytoplasmic and nuclear stiffness, a characteristic that may confer resistance to chemotherapeutic agents such as paclitaxel and radiotherapy (Xuan et al. 2018; Zhang et al. 2021). Although there is no universal threshold for defining drug‐resistant cells based solely on stiffness, these findings suggest that mechanical properties might serve as a prognostic marker for therapeutic responsiveness. A better understanding of how cell stiffness correlates with resistance to specific drug groups could provide rapid and cost‐effective personalized treatment strategies.

The regulation and sensing of cellular stiffness are emerging as critical factors in cancer diagnosis, prognosis, and treatment. While numerous studies have linked a softer mechanical phenotype to increased tumorigenicity, metastasis, and stemness, the relationship between stiffness and treatment resistance is complex and remains incompletely understood. Advances in measurement technologies and biophysical assays are making it increasingly feasible to incorporate cell mechanics into clinical decision‐making. Despite the need for further research to establish universal thresholds and understand drug‐specific responses, this field is a promising frontier for both biomarker discovery and therapeutic innovation.

## 5 Experimental Methods for Measuring Cell Mechanics

The mechanical properties of single cells‐stiffness, elasticity, and viscosity‐are central to structural integrity, function, and crosstalk with the microenvironment. They regulate migration, differentiation, and mechanotransduction, making quantitative assessment crucial for both physiology and pathology (Discher et al. 2005; Moeendarbary and Harris 2014; Janmey and Miller 2011). These properties are typically summarized by the elastic modulus (stiffness/resistance to deformation) and viscosity (dissipation of mechanical energy over time) (Suresh 2007). Because cells are viscoelastic‐exhibiting both fluid‐like and solid‐like behavior‐their responses are described with models such as spring–dashpot, power‐law viscoelasticity, and poroelasticity (Moeendarbary et al. 2013; Chaudhuri et al. 2020).

An extensive toolbox now probes cell mechanics across spatial and temporal scales. Broadly, methods fall into optical, magnetic/force‐based, microfluidic, cantilever/Micro‐Electro‐Mechanical Systems (MEMS), and hybrid categories (Suresh 2007). Optical approaches‐including acousto‐holography, optical tweezers, and laser‐induced photothermal vibration‐enable non‐contact measurements using light and radiation forces. Magnetic and other force‐based tools, such as magnetic tweezers and automated microhand systems, apply controlled loads to quantify deformation (Tanase et al. 2007). Microfluidic platforms (e.g., deformability cytometry and impedance sensors) deliver high‐throughput mechanical phenotyping suited to translational workflows. Cantilever‐ and MEMS‐based techniques‐including nanomechanical resonators and MEMS weighing cells‐offer micro‐ to nanoscale precision (Burg et al. 2007; Rajagopalan and Saif 2011). Hybrid schemes combine modalities to enhance accuracy and biological insight; for example, fluorescence‐lifetime imaging paired with AFM links biochemical states to biomechanical readouts (Efremov et al. 2023). Each technique entails trade‐offs among spatial resolution, throughput, invasiveness, and suitability for live‐cell measurements (Darling and Di Carlo 2015). Across techniques, the apparent modulus is not directly comparable unless key protocol variables are aligned. In particular, shallow indentation tends to emphasize the actin‐rich cortex (stiffer readouts) whereas larger deformations integrate cytoplasmic–nuclear mechanics; additionally, the loading rate/frequency controls the viscoelastic response (high rate/frequency appears stiffer) (Wu et al. 2018; Hecht et al. 2015; de Sousa et al. 2020). Finally, adherence on rigid 2D substrates elevates actomyosin prestress via focal adhesions, whereas suspension or compliant 3D contexts reduce prestress and commonly lower the apparent stiffness (Discher et al. 2005; Solon et al. 2007; Pelham and Wang 1997). Therefore, the method‐specific subsections below should be interpreted in light of indentation depth/scale, loading rate/frequency, and culture configuration (Table 1).

**Table 1:** Comparison of cell stiffness measurement techniques.

Platform	Perturbation/Contact	Spatial resolution (type + value + units)	Sample compatibility	Absolute E?	Model/Assumption	Example use	References
AFM nanoindentation	Indentation, contact	contact: depth‐dependent (tip radius < 30 nm)	Adherent cells; surface‐accessible samples	Yes (model‐based)	Hertz/Sneddon‐type fitting; requires probe geometry, ν	Cancer mechanophenotyping	(Haase and Pelling (2015))
RT‐DC (real‐time deformability cytometry)	Shear in microchannel, contact‐less	cell‐scale: 20 × 20 µm channel (also 30 × 30 µm for larger cells)	Whole blood, suspension cells	Indirect (model‐dependent)	Hydrodynamic stress model + linear elasticity (isoelasticity lines)	Hematology; acute myeloid leukemia (AML) blasts	(Otto et al. (2015))
Brillouin microscopy	Optical, contact‐less	voxel: 0.5 × 0.5 × 2 µm³; earlier: 6 × 6 × 60 µm³	Live cells/tissue; organoids	Proxy (Brillouin shift → longitudinal modulus)	Photoelastic relation; water/refractive index (RI) sensitivity	Brain tumor/cornea biomechanics	(Scarcelli and Yun (2008); Scarcelli et al. (2015))
Optical tweezers	Optical trap, contact‐less	tracking: 3.4 Å practical limit (sub‐nm); trap is diffraction‐limited	Bead/cell in suspension; membrane tethering	Model/calibration dependent	Trap stiffness calibration; position detection limits	Single‐molecule and cell mechanics	(Neuman and Block (2004))
Magnetic twisting cytometry (MTC/3D‐MTC)	Magnetic torque via bead (local)	probe: 4.5 µm bead; tracking noise < 5 nm	Adherent cells (ligand‐bead)	Model dependent	Bead rotation/translation vs time/frequency	Breast cell lines; mechanotransduction	(Zhang et al. (2017))
Microfluidic constriction arrays	Hydrodynamic constriction, contact (walls)	cell‐scale: constriction 5 µm (W) × 9 µm (H)	Cells in suspension (incl. trypsinized adherent cells)	Indirect (model‐dependent)	Entry/transit mechanics; power‐law rheology‐based inference	High‐throughput mechanophenotyping	(Lange et al. (2015))
Micropipette aspiration	Contact + negative pressure	probe: pipette radius ~2 µm; tracking: edge accuracy ~25 nm	Suspension cells	Yes (model‐based)	Continuum models (Laplace‐type and elastic solid approximations)	Leukocytes; red blood cells (RBCs)	(Hochmuth (2000))
Particle‐tracking microrheology (video tracking)	Passive (thermal), tracking‐based	tracking: 10 nm (xy); 150 nm (z); static error ~10 nm	Beads in cells/tissues; adherent or embedded samples	Proxy (complex shear modulus (G*) from mean squared displacement (MSD) via microrheology assumptions)	Subpixel localization; generalized Stokes‐Einstein relation (GSER) ‐type conversion in microrheology	Cytoplasm/ECM viscoelasticity mapping	(Crocker and Grier (1996))
Digital/acousto‐holography	Acoustic stimulation + holographic imaging, contact‐less	voxel: lateral 644–660 nm; axial ~45 nm	Single/multiple cells; stiffness map reconstruction	Model & calibration required	Viscoelastic/elastic inversion + system calibration	Whole‐cell stiffness maps (HCT116; CTC‐mimic)	(Varol et al. (2022))
MEMS micromechanical pads/cantilevers (traction‐type)	Contact (cell–pad)	contact: pad area 4–25 µm²	Adherent cells	Yes (device model)	Beam mechanics; optical deflection readout	Traction forces in migrating cells	(Rajagopalan and Saif (2011))

*Note*: The table provides an overview of different methods used to measure cellular mechanical properties, highlighting their working principles, advantages, and limitations. Techniques vary in terms of resolution, throughput, ease of implementation, and biological relevance, making them suitable for different research applications in mechanobiology, cell mechanics, and disease diagnostics. “Spatial resolution” is method‐dependent and is reported here as the smallest directly sampled spatial unit defined by each platform's operating principle: voxel (optical 3D sampling volume), contact (mechanical contact footprint/area), probe (physical probe size such as bead or pipette), tracking (localization precision), or cell‐scale (global deformation constrained by channel/constriction dimensions). Values are taken from the cited primary sources; when a single numeric resolution is not uniquely defined (e.g., AFM indentation), the entry is reported as depth‐/geometry‐dependent.

### 5.1 Optical‐Based Methods for Cell Stiffness Measurement

Optical methods have become indispensable for probing single‐cell mechanics, offering non‐contact force application, high spatial resolution, and real‐time readout. They provide quantitative insight into stiffness, elasticity, and viscosity‐key parameters underlying migration, differentiation, and disease progression. While early light‐microscopy studies laid the foundation, major advances came with lasers, interferometry, and computational imaging. Modern quantitative phase imaging, holography, and optical tweezers now achieve nanometer‐level sensitivity with minimal invasiveness. Compared with contact‐based techniques such as AFM or micropipette aspiration, optical approaches enable label‐free, dynamic mapping of intracellular mechanics, ideal for fragile structures like the cytoskeleton and nucleus (Moeendarbary et al. 2013). Recent progress in ultrafast imaging, AI‐driven analysis, and microfluidic integration further increases scalability for both mechanobiology and translational applications (Nguyen et al. 2022b; Chen et al. 2022; Herbig et al. 2022).

#### 5.1.1 Optical Tweezers and Optical Trapping Techniques

**Figure 4:**
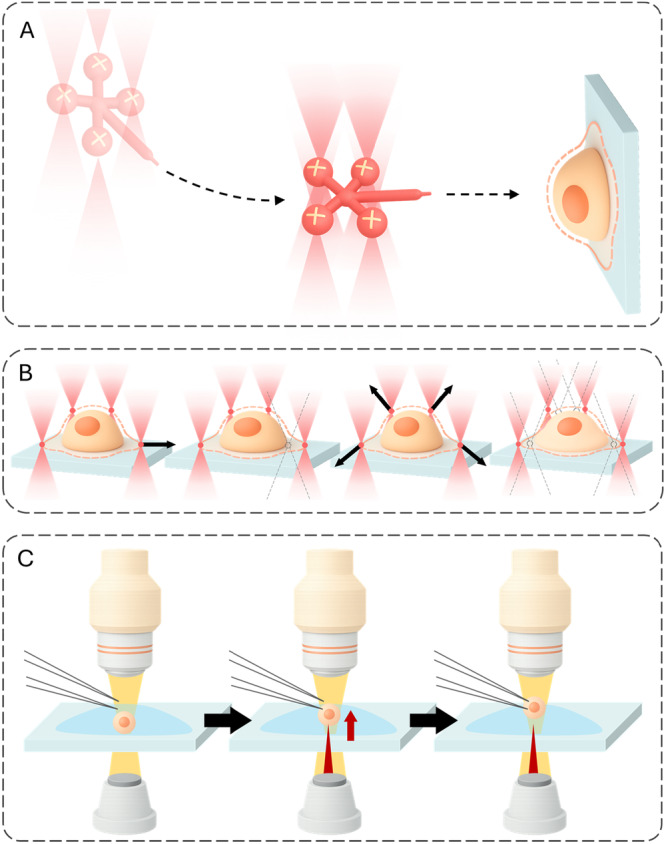
(A) Sample preparation and manipulation process using microtools (red structures) is shown. After pipetting into the sample well, the microtools are aligned with the target cell. Red crosses indicate optical trap beam positions, dashed arrows represent stage movement, and red light illustrates the optical trap activation (Grexa 2020). (B) Schematic illustration of multi‐trap optical stretching configurations. Optical trap foci (red dots) and the trapping‐beam geometry (pink cones) are used to impose different deformation modes on an adhered cell. The orange dashed outline indicates the reference (undeformed) cell boundary, while the solid outline represents the deformed shape. In the first two sketches, asymmetric stretching is illustrated by applying a single‐sided/single‐axis pull. In the third sketch, symmetric stretching is achieved by translating opposing trap pairs outward simultaneously. The dotted gray lines (dotted lines) in the third sketch denote the intended trap‐translation direction, that is, the stretching (trap displacement) axis, and thus indicate the direction along which force is applied. The rightmost sketch illustrates that the deformation direction can be probed by applying translations along alternative axes (multi‐axial or sequential stretching) (Merola et al. 2017). (C) Optical tweezer‐assisted manipulation process (Kawakami et al. 2022): (i) Initial configuration showing the target object within the optical trap, (ii) the target object is moved to the focal plane of the laser by the automated stage, (iii) optical tweezers elevate the target object to match the height of the end‐effector and the end‐effector grasps the target object.

Optical tweezers, introduced by Ashkin in the 1980s, use a tightly focused laser to apply radiation‐pressure forces on dielectric particles, enabling precise piconewton‐scale manipulation. This precision makes them powerful tools for studying cytoskeletal mechanics, membrane tension, and cell elasticity. Force‐feedback implementations have improved accuracy; for instance, Rodenburg et al. (2025) used optically trapped beads with feedback control to quantify deformation and stiffness in real time (Rodenburg et al. 2025). Coupling tweezers with digital holography extends this capability to red blood cell mechanics: wavefront distortions arising from RBC deformation can be analyzed to estimate membrane stiffness and elasticity (Merola et al. 2017) (see Figure 4B), providing a sensitive readout for hematologic disorders.

Kawakami et al. (2022) developed an automated microhand platform for precise, reproducible stiffness measurements. The system combines dual plate‐shaped end‐effectors, a microforce sensor, optical tweezers, and integrated imaging (Kawakami et al. 2022). Optical tweezers lift and align the cell, while the microhand applies controlled compression to compute stiffness (Figure 4C). Plate‐type end‐effectors provide a larger, well‐defined contact area than needle geometries, improving whole‐cell deformation accuracy. Automated positioning reduces operator dependence and supports consistent results across users. The method has been demonstrated on HeLa, C2C12, HEK293A, and HepG2 cells, showing high accuracy, speed, and reliability.

#### 5.1.2 Computational Optical Flow‐Based Stiffness Mapping

Optical flow based stiffness mapping estimates intracellular mechanical contrast from time‐lapse fluorescence or phase‐contrast image sequences. By constraining optical flow with appropriate mechanical models, this computational approach yields relative stiffness distributions without direct force application. Kesenci et al. (2024) introduced a methodology that enables high‐throughput, label‐free mechanical phenotyping, particularly useful for live‐cell monitoring and biomechanics studies where physical perturbation is undesirable (Kesenci et al. 2024).

### 5.2 Magnetic‐ and Force‐Based Methods for Cell Stiffness Measurement

Magnetic‐ and force‐based approaches probe cell mechanics by applying controlled loads and measuring the resulting deformation or reaction forces. Using magnetic fields or mechanical actuators, these methods characterize cellular responses under defined stimuli. Compared with contact‐based techniques such as AFM or micropipette aspiration, magnetic strategies offer remote actuation, tunable forces, and higher throughput, making them suitable for live‐cell studies under physiological conditions.

**Figure 5:**
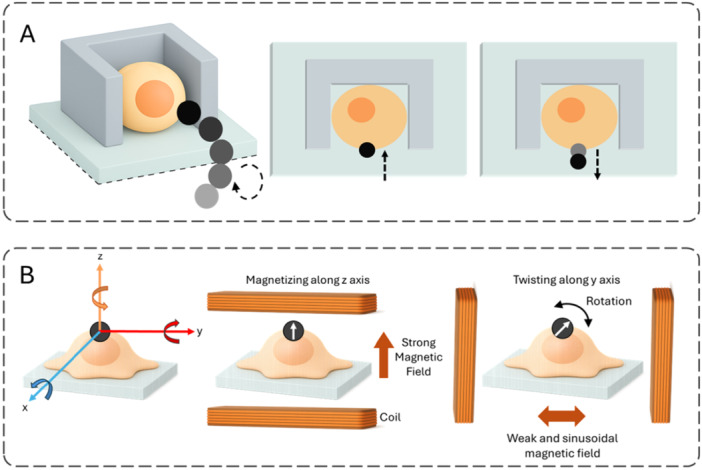
(A) Magnetic bead–cell contact procedure (Tang 2021b): Magnetic beads are navigated within a microfluidic trapping unit to approach the cell. Once in contact, the magnetic bead exerts controlled forces to deform the cell surface, enabling high‐resolution stiffness measurements using confocal imaging. (B) Application of controlled shear stress with 3D‐MTC (Zhang et al. 2017): A ligand‐coated ferromagnetic bead bound to the cell surface is first magnetized along the z‐axis by a strong magnetic pulse and subsequently rotated by applying a weak sinusoidal magnetic field along a perpendicular axis. This generates shear stresses that deform the cell, enabling quantitative stiffness characterization.

Magnetic force application originated with early work using ferromagnetic particles for biological manipulation and later advanced with magnetic twisting cytometry (MTC), which applies torque through bound beads and enables multi‐axial loading with real‐time imaging (Zhang et al. 2017) (Figure 5B). In parallel, micromanipulation matured with automated microhands and integrated microforce sensors (Kawakami et al. 2022), improving precision and throughput.

A major advance was coupling magnetic tweezers with microfluidics to control bead–cell contact, force magnitude, and timing in arrayed formats (Tang 2021b). Steering magnetic beads within microchannels allows controlled deformation and efficient, reproducible measurement (Figure 5A). This hybridization addresses earlier limitations in throughput and force consistency, benefiting mechanotransduction studies, regenerative medicine, and cancer diagnostics.

While conventional tweezers typically provide single‐axis actuation, 3D‐MTC offers multi‐axial control, enabling detailed analysis of viscoelasticity and cytoskeletal remodeling. Combined with confocal fluorescence microscopy, it supports simultaneous mechanical stimulation and structural readout, yielding insight into mechanotransduction pathways. Even as some methods infer stiffness indirectly, automated dual‐end‐effector microhands now deliver direct, reproducible measurements with minimal operator variability‐well suited for applications such as drug testing and diagnostic screening where consistency and precision are paramount (Kawakami et al. 2022).

### 5.3 Microfluidics‐Based Methods for Cell Stiffness Measurement

Microfluidics has transformed single‐cell mechanophenotyping by enabling high‐throughput, label‐free, and automated stiffness measurements. Whereas AFM and micropipette aspiration offer precision but limited throughput, microfluidic platforms leverage hydrodynamic forces, controlled flow, and real‐time readouts to induce and quantify deformation at scale. From these responses, parameters such as shear modulus, elastic modulus, and viscosity can be extracted rapidly, supporting applications in diagnostics, mechanobiology, and drug screening. Practical deployment often requires tuning channel geometry, surface coatings, and flow rates for specific cell types, which can introduce protocol variability across laboratories.

Advances from passive‐deformation assays to platforms with high‐speed imaging, impedance sensing, and machine learning have greatly improved precision and throughput. Modern systems now enable population‐scale analyses, processing thousands of cells per second, while deep learning streamlines data interpretation, though requiring robust computation and automation.

#### 5.3.1 High‐Throughput Shear Modulus Measurement of Red Blood Cells

**Figure 6:**
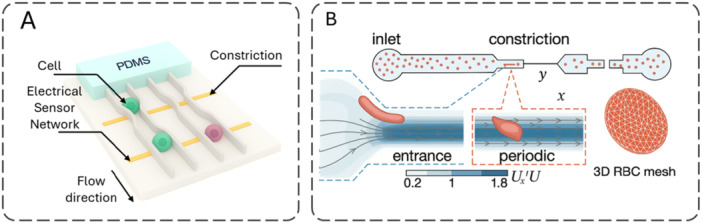
(A) Polydimethylsiloxane (PDMS)‐based microfluidic device integrated with a coded electrical sensor network. Each sensor pair timestamps cells just before and after they traverse a constriction, enabling the calculation of cell transit time as an indicator of mechanical properties (Asmare et al. 2025). (B) Design of the microfluidic constriction channel (top) and corresponding finite element simulation setup (bottom). The computational model includes entrance and periodic regions with defined boundary conditions, along with a 3D red blood cell (RBC) mesh used to simulate deformation and flow behavior within the constriction (Saadat et al. 2020).

RBC deformability is a central biomarker for hematologic disorders, malaria, and sickle cell disease. To overcome the throughput limits of optical trapping and micropipette aspiration, microfluidic devices use variable constriction channels to subject RBCs to defined shear while high‐speed imaging tracks deformation dynamics (Saadat et al. 2020) (Figure 6B). From these trajectories, shear modulus distributions are quantified at scale‐enabling rapid, label‐free blood diagnostics and supporting personalized screening across large cohorts.

#### 5.3.2 Impedance‐Based Cytometry for Real‐Time Stiffness Assessment

As an imaging‐free complement to optical methods, impedance cytometry quantifies stiffness proxies via electrically encoded transit signatures. Multiplexed sensor pairs embedded in PDMS channels timestamp cells at entry and exit of constrictions, yielding transit times that correlate with mechanical properties, while signal amplitude separates size effects (Asmare et al. 2025) (Figure 6A). This architecture improves automation and speed, facilitating integration into lab‐on‐a‐chip workflows for immunoprofiling, regenerative medicine, and cancer cell recognition with minimal sample preparation.

#### 5.3.3 Deep Learning Driven Prediction of Cell Mechanical Properties

Machine learning further elevates throughput and accuracy by mapping deformation sequences directly to mechanical parameters. Gong et al. (2024) introduced a multi‐input convolutional neural network (MI‐CNN) that ingests time‐resolved contour data alongside temporal and positional metadata to predict elastic modulus or classify constitutive behavior. The model achieved an $R^{2}$ of 0.999 for stiffness estimation, surpassing traditional model‐based pipelines and enabling real‐time, AI‐assisted biomechanical screening at scale (Gong et al. 2024).

### 5.4 Cantilever‐ and MEMS‐Based Methods for Cell Stiffness Measurement

Cantilever‐ and MEMS‐based approaches have advanced single‐cell mechanics by combining label‐free operation, high precision, and nanoscale resolution. These methods use microfabricated structures whose bending, resonance, or strain directly report forces and deformations, yielding quantitative stiffness and elasticity. While AFM remains a benchmark for nanoscale force measurements, its optical alignment requirements, limited throughput, and system complexity constrain broader use. To address these limitations, recent work has optimized cantilever geometries and integrated on‐chip transduction, producing platforms that are more scalable and better suited to routine cell measurements.

**Figure 7:**
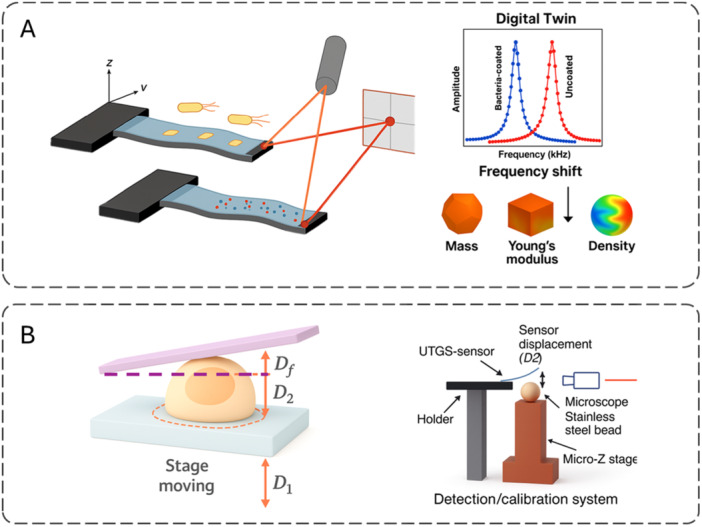
Schematic illustration of microcantilever‐based biosensing and ultrathin glass sheet (UTGS) cantilever measurement setup. (A) Microcantilever biosensing platform for detecting resonance frequency shifts caused by target binding events (Bhattacharya et al. 2024). The system uses a digital twin model to correlate frequency shifts with changes in the target's physical properties, including mass, stiffness, and density. (B) Shape‐modified UTGS cantilever system for single‐cell stiffness measurement (Yuan 2024). The setup involves positioning the cantilever sensor in contact with the sample and using a controlled stage movement for measurement. A stainless steel bead and micro‐Z stage are employed for calibration.

A notable development is the shape‐modified UTGS cantilever, which overcomes limitations of conventional silicon devices. By integrating a strain‐gauge sensor on a transparent glass sheet, the UTGS system enables direct electrical readout while preserving optical access and chemical stability (Figure 7B) (Yuan 2024). Its flexibility and simpler fabrication improve adaptability for probing soft, delicate samples, including living cells and thin tissues.

Parallel advances in nanomechanical resonators allow simultaneous assessment of adsorbed‐layer mass and stiffness. Bhattacharya et al. (2024) introduced a framework separating frequency shifts due to mass versus stiffness changes, enabling accurate measurements of proteins, lipid bilayers, and living bacteria under ambient conditions (Figure 7A).

### 5.5 Acoustic Stimulation‐Based Methods

Acoustic stimulation‐based approaches quantitatively assess cell mechanics by applying a controlled, oscillatory excitation to the cell, typically via piezoelectric actuators or acoustofluidic fields. Unlike static indentation assays, these methods inherently enable dynamic measurements and, when paired with appropriate material models, support frequency‐domain characterization and the extraction of viscoelastic properties. Furthermore, their straightforward integration into microfluidic architectures without requiring direct physical contact makes them attractive for standardized mechanophenotyping workflows.

To capture these dynamic responses, digital holography captures phase shifts to perform quantitative phase imaging (QPI). While phase primarily reflects optical path length, set by thickness and refractive index, its temporal evolution under controlled perturbation can report mechanical properties with high resolution and without physical contact (Marquet et al. 2014; Huang and Cao 2024).

**Figure 8:**
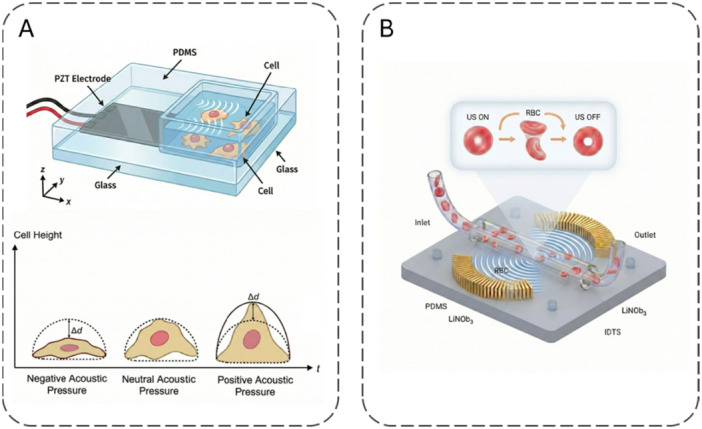
(A) Bulk‐acoustic‐wave (BAW) actuation chip and acoustically induced cell deformation readout. Cells are cultured inside a PDMS microfluidic chamber mounted on a glass substrate and actuated by a bonded PZT transducer/electrode that generates low‐intensity BAW excitation (∼1 kHz). The resulting whole‐cell deformation is monitored with interferometric/holographic imaging to quantify time‐resolved height (thickness) changes and infer stiffness‐related mechanical response. The lower schematic illustrates representative deformation states under negative, neutral, and positive acoustic pressure, where the peak‐to‐peak thickness modulation is denoted by Δd (Varol et al. 2022). (B) Schematic illustration of cell deformation induced by the acoustic squeezer. Standing surface acoustic waves generated by interdigitated transducers (IDTs) on a LiNbO₃ substrate act across a PDMS microfluidic channel, deforming red blood cells as they pass through the actuation region (US ON) and allowing relaxation/recovery when the ultrasound is switched off (US OFF) (Zhou et al. 2025).

A notable example in this category is the acousto‐holographic stiffness mapping method developed by Varol et al (Varol et al. 2022). This technique combines periodic acoustic pressure waves with digital holographic microscopy to generate high‐speed, whole‐cell stiffness maps (see Figure 8A). The authors highlighted its advantages over AFM and optical trapping in terms of throughput and the reduction of contact‐induced artifacts, and demonstrated mechanical discrimination between epithelial HCT116 cells and their TGF‐β‐treated CTC‐mimicking counterparts. Rather than characterizing the cell with a single average modulus, this approach provides spatially resolved stiffness distributions, which are critical for capturing subcellular heterogeneity.

In a more recent study, Zhou et al. introduced an “acoustic squeezer” concept for multiparameter mechanical assessment in RBCs (see Figure 8B), extracting Young's modulus and viscosity from the acoustic deformation response under controlled excitation (Zhou et al. 2025). Such platforms are often discussed within the broader framework of deformability cytometry, where acoustic fields generate controlled stresses on cells in flow. Consequently, high‐throughput and operator‐independent mechanical screening becomes possible, particularly when model‐based fitting is used to improve the interpretability of the acquired deformation signatures.

Another vibration‐oriented approach was reported by Abdioğlu et al. using a vibration‐encoded in‐line Mach‐Zehnder holography system (Abdioglu et al. 2026). In this study, holograms acquired over a single vibration cycle were processed to extract the static phase, modulation depth, and phase lag information. By linking these observables to a Kelvin‐Voigt model, storage and loss modulus maps were generated simultaneously. The method was validated on calibrated polyacrylamide beads and tested on adherent MCF‐7 breast cancer cells, demonstrating that cellular viscoelasticity can be spatially mapped under periodic excitation.

### 5.6 Hybrid and Multi‐Functional Approaches for Cell Stiffness Measurement

Traditional methods such as AFM, micropipette aspiration, and optical tweezers have been central to quantifying cell stiffness and viscoelasticity. Yet each offers only a partial view: some sacrifice resolution, others underrepresent cell–matrix interactions, and many are limited in throughput. To address these gaps, hybrid and multifunctional platforms now combine complementary readouts to deliver a fuller picture under physiologically relevant conditions.

One example is a multifunctional sensor that measures both cell traction forces and matrix stiffness, allowing simultaneous tracking of cell‐generated forces and ECM remodeling. This moves beyond static stiffness measurements to capture how cells push, pull, and reshape their surroundings‐capabilities that are especially useful in tissue engineering and cancer biology, where altered cell–ECM coupling can signal differentiation state or disease progression.

Although the cytoskeleton is a primary determinant of mechanics, membrane properties and intracellular viscosity also contribute‐factors often missed by single‐modality assays. Pairing fluorescence‐lifetime imaging microscopy (FLIM) with AFM links plasma‐membrane microviscosity to cellular stiffness, providing both mechanical and biochemical context in a single experiment (Efremov et al. 2023).

As techniques proliferate, comparative studies are essential for matching tools to questions and sample types. Head‐to‐head evaluations of force‐application versus force‐sensing strategies help clarify strengths and limitations across biological systems. In parallel, automated, data‐driven analysis pipelines are improving interpretability and precision‐particularly important in high‐throughput settings where consistent, unbiased readouts are critical.

## 6 Conclusion

Cytoskeletal components, cell‐cell and ECM interactions, and the dynamic nature of the tumor microenvironment are key parameters influencing single‐cell stiffness. These biophysical and biochemical factors shape the mechanical phenotype of cancer cells, directly impacting invasion, migration, metastatic dissemination, and therapeutic resistance. Variations in stiffness, whether as softening or stiffening, provide valuable insights into tumor biology and therapeutic response, enabling a nuanced understanding of how cancer cells react to biomechanical and biochemical cues.

Understanding single‐cell mechanics is therefore a critical step toward the development of mechanics‐based diagnostic and therapeutic approaches, early cancer detection, the identification of novel therapeutic targets, and the personalization of treatment strategies. A deeper appreciation of the molecular underpinnings, such as the integrin FAK‐YAP/TAZ axis, mechanosensitive ion channels like Piezo1, and cytoskeletal remodeling, offers opportunities for targeted interventions capable of modulating tumor mechanics to inhibit disease progression.

To characterize these changes in stiffness, researchers have developed a broad range of complementary techniques that differ in scale, sensitivity, throughput, and complexity. Contact‐based methods such as AFM, micropipette aspiration, and MTC provide high‐resolution, localized force measurements, excelling at probing viscoelastic properties and cytoskeletal reorganization. Non‐contact optical strategies, including optical tweezers, optical stretching, Brillouin microscopy, and acousto‐holography, minimize mechanical artifacts while enabling label‐free, real‐time imaging of cells under dynamic conditions. High‐throughput microfluidic approaches allow rapid mechanical phenotyping of thousands of cells per second under well‐controlled mechanical stresses, making them particularly suited for identifying invasive or drug‐resistant subpopulations. Integrating imaging modalities such as high‐speed fluorescence microscopy or digital holography into these platforms enables simultaneous biochemical and biomechanical profiling.

While each method has inherent trade‐offs in terms of throughput versus resolution, mechanical artifact versus non‐contact probing, or instrumentation complexity versus accessibility, ongoing advances in instrumentation, automation, and computational analytics are steadily overcoming these barriers. Synergizing multiple approaches, for example combining microfluidics with optical and force‐based tools, and incorporating artificial intelligence (AI)‐driven, real‐time data analysis will further refine our ability to map, quantify, and interpret cell stiffness. Equally important is the establishment of standardized measurement protocols, inter‐laboratory calibration, and regulatory validation to ensure reproducibility and comparability of mechanical biomarkers across platforms.

Looking forward, longitudinal mechanophenotyping during therapy could reveal dynamic biomechanical trajectories predictive of treatment response or relapse, adding a powerful dimension to patient monitoring. The integration of stiffness data with genomic, transcriptomic, and proteomic profiles, known as mechano‐omics, will yield comprehensive biophysical signatures predictive of metastatic potential, drug resistance, and therapeutic outcome. Organ‐on‐a‐chip and 3D bioprinted tumor models replicating physiologically relevant microenvironments will bridge the gap between in vitro assays and in vivo complexity, enabling real‐time mechanophenotyping under dynamic biochemical and mechanical stimuli.

In the clinical context, miniaturization, automation, and cost reduction of these technologies, coupled with liquid biopsy‐based mechanical profiling, could enable minimally invasive diagnostics and disease monitoring. As mechanobiology converges with bioengineering, computational modeling, and artificial intelligence, mechanical biomarkers are poised to enter the clinical arena, creating a new paradigm in precision oncology where stiffness‐based phenotyping informs early detection, risk stratification, and individualized therapeutic strategies.

Cytoskeletal components, cell‐cell and ECM interactions, and the dynamics of the tumor microenvironment are key parameters influencing single‐cell stiffness. These biophysical and biochemical factors shape the mechanical properties of cancer cells, directly affecting critical processes such as invasion, migration, and metastasis. Changes in cell stiffness provide valuable insights into cancer progression and therapeutic response, enabling a more detailed analysis of how cancer cells react to biomechanical and biochemical signals.

In this context, understanding single‐cell mechanics is a crucial step toward the development of mechanics‐based diagnostic and therapeutic approaches, early cancer detection, the identification of novel therapeutic targets, and the advancement of personalized treatment strategies. A deeper understanding of cell mechanics can contribute to a more comprehensive knowledge of cancer biology, ultimately facilitating the development of next‐generation therapeutic approaches.

To characterize these changes in stiffness, researchers have devised a range of techniques that differ in scale, sensitivity, throughput, and complexity. Contact‐based methods such as AFM, micropipette aspiration, and magnetic twisting cytometry remain essential for high‐resolution, localized force measurement. They excel at probing viscoelastic properties and cytoskeletal reorganization but can be time‐consuming and relatively low‐throughput. Non‐contact optical strategies, including optical tweezers, optical stretching, Brillouin microscopy, and acousto‐holography, reduce mechanical artifacts while allowing label‐free, often real‐time imaging of cells under dynamic conditions. Moreover, acousto‐holography stands out for its capacity to provide high‐resolution volumetric stiffness maps, which can reveal subtle subcellular mechanical heterogeneities pertinent to processes such as nuclear deformation and stemness. Nonetheless, these techniques may require advanced instrumentation and often need sophisticated modeling and data analysis to interpret measurements accurately.

High‐throughput microfluidic approaches complement these platforms by enabling the rapid screening of thousands of cells per second under well‐controlled shear, compression, or extensional flow fields. In a clinical or translational setting, these microfluidic methods carry significant potential as they offer population‐level insights into mechanical phenotypes, assisting in the identification of highly invasive or drug‐resistant cell subsets. Integrating imaging modalities‐such as high‐speed fluorescence microscopy or digital holography‐within microfluidic channels further allows concurrent biochemical and mechanical profiling, which is valuable for dissecting the multifaceted mechanisms of cancer progression.

Taken together, these diverse measurement methodologies underscore the promise of stiffness‐based phenotyping as part of a new wave of cancer diagnostics and therapeutics. While each method has inherent trade‐offs‐whether in throughput versus resolution, mechanical artifact versus non‐contact probing, or specialized equipment versus ease of adoption‐ongoing improvements in instrumentation, automation, and computational analytics are steadily overcoming these barriers. Looking ahead, synergizing multiple approaches (e.g., combining microfluidics with optical and force‐based tools) and leveraging machine learning for real‐time data processing will likely refine our ability to map, quantify, and interpret cell stiffness. In parallel, translating these findings into clinical workflows‐where rapid, reliable mechanical markers can aid in early detection, patient stratification, and treatment monitoring‐remains an important frontier. A deeper understanding of the biomechanical landscape in cancer promises to transform current paradigms in oncology, offering more personalized and effective strategies for combating this disease. In conclusion, the dual role of cell stiffness in cancer biology, both as a biomarker and as a mediator, may pave the way for the development of novel diagnostic and therapeutic strategies.

The dynamic and heterogeneous nature of cancer necessitates a shift toward single‐cell‐level investigations to uncover the mechanical factors driving tumor progression. As mechanobiology becomes increasingly central to understanding tumor behavior, high‐resolution, label‐free, and real‐time measurement technologies will play a critical role. The convergence of micro‐ and nanotechnologies, advanced imaging systems, and artificial intelligence is expected to revolutionize how single‐cell mechanical properties are measured and interpreted. Emerging biosensor platforms that integrate microfluidics with optical or electrical detection modalities are poised to provide robust, clinically translatable tools for rapid mechanophenotyping. Simultaneously, the evolution of established methods such as AFM, optical tweezers, and real‐time deformability cytometry is advancing toward higher throughput, improved sensitivity, and compatibility with clinical applications. A major future direction involves the multimodal integration of mechanical data with genomic, transcriptomic, and proteomic profiles to develop comprehensive biophysical signatures of cancer cells. This interdisciplinary approach, often referred to as mechano‐omics, will enable the identification of mechanical biomarkers predictive of metastatic potential, drug resistance, and treatment response. As the tumor microenvironment is increasingly recognized as a critical regulator of cell mechanics, future studies must focus on replicating physiologically relevant conditions using organ‐on‐a‐chip technologies and 3D bioprinted models. These platforms will facilitate real‐time monitoring of cellular mechanics under dynamic biochemical and mechanical stimuli, closing the gap between in vitro experimentation and in vivo complexity. In the clinical context, the miniaturization, automation, and cost reduction of these technologies will be essential for widespread adoption. Liquid biopsy samples, combined with rapid mechanical profiling, offer the potential for minimally invasive cancer diagnostics and disease monitoring. Over the next decade, mechanical phenotyping is expected to play a key role not only in diagnosis but also in prognostication and the personalization of therapeutic strategies. In summary, as technological and analytical advancements converge, the integration of mechanical biomarkers into precision oncology could establish a new paradigm in cancer management‐enabling earlier detection, improved risk stratification, and more effective, individualized treatment approaches.

## Author Contributions

**Merve Sevgi:** writing – review and editing, writing – original draft, visualization, conceptualization, investigation. **Yağmur Işık:** conceptualization, investigation, writing – original draft, visualization, writing – review and editing. **Caner Karaca:** investigation, conceptualization, writing – original draft. **Esmahan Çağlar:** writing – original draft, investigation. **Hasan Berkay Abdioğlu:** investigation, writing – original draft. **Ferican Zendel:** investigation, writing – original draft. **Yasemin Başbınar:** investigation, writing – original draft, conceptualization. **Hüseyin Üvet:** conceptualization, methodology, funding acquisition, writing – review and editing.

## Ethics Statement

The authors confirm that all methods were performed in accordance with the relevant guidelines and regulations. For studies involving human cell lines, no ethical approval was required as the cell lines were commercially obtained and did not involve primary human samples.

## Conflicts of Interest

The authors declare no conflicts of interest.

## Data Availability

Data sharing not applicable to this article as no datasets were generated or analyzed during the current study.
